# Retrospective Radiographic Study of Degenerative Joint Disease in Cats: Prevalence Based on Orthogonal Radiographs

**DOI:** 10.3389/fvets.2020.00138

**Published:** 2020-03-31

**Authors:** Taro Kimura, Sayaka Kimura, Junichi Okada, Sayaka Suzuki, Taku Kitanaka

**Affiliations:** ^1^Kimura Animal Hospital, Tokyo, Japan; ^2^Department of Biochemistry, Nihon University, Kanagawa Ward, Japan

**Keywords:** cat, degenerative joint disease, diagnosis, orthogonal radiograph, osteoarthritis

## Abstract

Feline degenerative joint disease (DJD) has been reported worldwide. Radiographic evidence, including that from single-plane radiographs, has been used for diagnosis in these reports, though orthogonal radiographs are generally required to diagnose DJD. However, more orthogonal radiographs are required for diagnosis. In this study, we investigated how many orthogonal radiographs are necessary to diagnose feline DJD among domestic short-haired cats. We analyzed the data from 101 cats for which the owners requested screening for arthritis. Orthogonal radiographs of appendicular and intervertebral joints were taken from the chest to the caudal side. Radiographs were then reviewed and graded by severity of DJD in each joint. Radiographic evidence of appendicular DJD was detected in 74.26% of 101 cats, of which 40.59% had intervertebral DJD (typically of the lumbosacral joint). Appendicular DJD was most common in elbow joints. Lameness was recognized by an owner of two cats and was diagnosed by a veterinarian in two cats. No obvious pain was detected on palpation in any cats with appendicular osteoarthritis, but lumbosacral DJD was associated with back pain in seven cases. Aging was associated with radiographic evidence of DJD: radiographic evidence of DJD was observed in most older domestic short-haired cats. Most cases without lumbosacral DJD had no obvious symptoms. As the lifespan of cats increases due to better lifestyles, diet, and medical treatment, lumbosacral DJD, which is more likely in older cats, may become an increasingly important clinical problem. In addition, orthogonal radiograph must be taken to make diagnosis for appendicular joint DJD especially hip and stifle joint.

## Introduction

Degenerative joint disease (DJD), which is used as a general term to describe both osteoarthritis (OA) and spondylosis deformans (SD), is common in older cats (1). Although there are some reports of DJD in cats, the problem has been considered less clinically significant than canine DJD (2–5).

In domestic cats, appendicular OA is identified radiographically by the presence of osteophytes, enthesophytes, increasing subchondral radio-opacity, soft tissue swelling, and mineralization of the articular tissue (2–5). In the axial skeleton, SD is identified radiographically by the presence of enthesophytes in fibrocartilaginous intervertebral joints (6). It is difficult to detect the region of DJD in cats by palpation, although this is generally useful in dogs (3).

Studies have attempted to determine the prevalence of DJD in domestic cats, given their large population, and a few reports have shown a correlation between age and this morbidity (3–5), although those reports have included diagnoses based on single-plane radiographs, from which it may be possible to miss the region of arthritis. Item generation and design testing of a questionnaire were both carried out to address these issues (7), and orthogonal radiographs have been used to gain evidence for evaluating DJD (8). However, there have been no reports to mention regarding the precise evaluation of feline DJD using orthogonal radiographs.

The aim of this retrospective study was to determine the prevalence of feline DJD among domestic short-haired cats precisely.

## Methods

### Study Subjects and Evaluation

The study was approved by the institutional animal care and use committee of Kimura Animal Hospital (KAH2014-004, KAH2015-003, and KAH2016-002), and informed consent was obtained from all owners.

We analyzed data from 101 domestic short-haired cats, aged older than 1 year, for which the owners requested screening of arthritis from May 2014 to February 2016. All cats underwent a general physical examination to confirm the appropriateness of sedation for taking radiographs and details of their age, sex, bodyweight, BCS, lifestyle, diagnosis, and any lameness were recorded. Orthogonal radiographs were then taken under sedation using a standard protocol. Orthopedic examination was performed when radiographic changes indicating severe joint problems were found.

**Figure 1:**
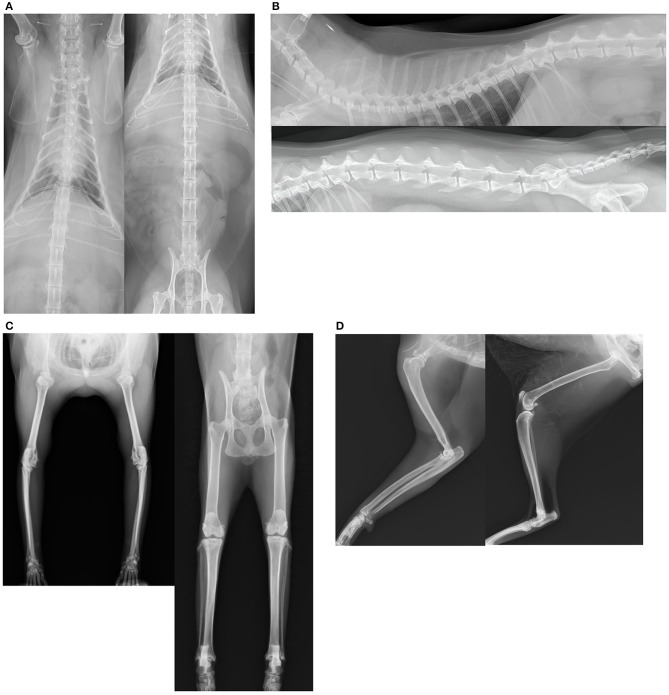
**(A–D)** Orthogonal radiographs were obtained to examine the intervertebral and appendicular joints.

All radiographs included two directions for vertebral and appendicular joints, excluding the cervical vertebrae and digital articulations, using appropriate equipment (Aero DR, Konica Minolta Japan, Inc., Hino, Japan). For forelimb evaluation, the shoulder, elbow, and carpus were included into a single plane in each of the mediolateral and anteroposterior directions. For hindlimb evaluation, the hip, stifle, and tarsus were included into a single plane in each of the mediolateral and ventrodorsal directions. For cranial spine evaluation, we include at least the fourth cervical vertebrae to the second lumber vertebrae into a single plane for each lateral position and ventrodorsal view, and at least the 10th thoracic vertebrae to the coccygeal vertebrae were included in the radiographs for evaluation of the caudal vertebrae (Figures 1A,B).

All the radiographs were evaluated by a Japanese Animal Hospital Association-certified surgeon. The diagnostic criteria for DJD in appendicular joints required visible osteophytes, soft tissue swelling, soft tissue mineralization, increasing subchondral radio-opacity, and enthesophytosis. Soft tissue swelling and increasing subchondral radio-opacity were graded as 0 (not visible) or 1 (visible). Osteophytes, enthesophytes, and soft tissue mineralization were graded on similar scales, from 0 to 3 (0, if the appearance was normal; 1, ≤ 1 mm in size; 2, >1–3 mm in size; and 3, >3 mm in size).

Ventrodorsal radiographs of the pelvis were used to evaluate hip joints. Hip dysplasia (HD) was identified when the acetabular depth was <50% that of the femoral head. The Norberg angle (NA) was measured as the angle formed by a line connecting the centers of both femoral heads and one drawn between the center of a femoral head and the craniodorsal rim of the acetabulum on the same side (9).

Enthesophytes and osteophytes in intervertebral joints were considered to indicate SD, and were graded on a scale from 0 to 3 (0, if the appearance was normal; 1, if they did not reach the vertebral endplate; 2, if they crossed over the vertebral endplate; and 3, if a bridge was formed between the vertebrae). The group in which SD or DJD was detected in the radiographs was regarded as an affected population, and the group in which no abnormal findings were detected was regarded as a healthy population. Congenital abnormalities of the vertebrae and appendicular joints were also recorded.

### Statistical Analysis

Data were compared between the healthy and affected populations, including appendicular OA and axial skeleton SD, to identify any differences in age, sex, bodyweight, BCS, or lifestyle. Correlation between symptoms and radiographic evidence was also investigated within the affected population. Statistical analyses were performed by Mann–Whitney *U*-tests, chi-squared tests, or Pearson's correlation coefficients, as appropriate, using StatMate V for Win&Mac Hybrid (ATMS Co., Ltd. Tokyo, Japan). Differences were considered significant when the *p* < 0.05.

## Results

We analyzed data from 101 domestic short-haired cats during the study period. This included seven females, 45 neutered females, 12 males, and 38 castrated males; 41 of the cats were allowed outdoors, and 60 cats lived indoors only. The median age (9.78 ± 5.77 years), BCS (3.44 ± 0.83), and weight (4.35 ± 1.51 kg) are shown in Table 1. Median age was significantly higher in both the OA group and SD group compared with the healthy group. There were correlations between sex and both appendicular joint OA and intervertebral joint SD, but there were no associations with body weight, BCS, and lifestyle.

**Table 1:** Results of general examination of all joints affected by appendicular osteoarthritis (OA) and spondylosis deformans (SD) (*n* = 101 cats).

	**Appendicular OA-affected cats**	**OA-free cats**	**SD-affected cats**	**SD-free cats**
Number of group	75	26	41	60
Age	11.63 ± 5.15 *	4.36 ± 3.93	14.24 ± 3.89*	6.73 ± 4.89
Body condition score (BCS)	3.45 ± 0.84	3.45 ± 0.85	3.30 ± 0.68	3.53 ± 0.85
Body weight (kg)	4.35 ± 1.51	4.15 ± 1.45	3.99 ± 1.20	4.58 ± 1.59
*Mann-Whitney ***U*****-test** ***P*** **<** **0.05**				
**Sex**		**Chi-squared test** ***P*** **<** **0.05**		**Chi-squared test** ***P*** **<** **0.05**
Neutered female	40/75	5/26	24/41	22/60
Female	5/75	2/26	1/41	6/60
Castrated male	27/75	11/26	15/41	23/60
Male	3/75	8/26	1/41	10/60
Indoor life	42/75	18/26	25/41	35/60

The number of affected appendicular joints and intervertebral joints is summarized in Figures 1C,D. There were 1,212 appendicular joints and 2,102 intervertebral joints visible on orthogonal radiograph images. Of the 101 cases, 75 cats (74.26%) had radiographic evidence of OA in appendicular joints, and 41 cats (40.59%) had radiographic evidence of SD. OA of the hip joint (28 joints) and meniscal calcification at the stifle joint (15 joints) could only be detected on cranio-caudal radiographs. Radiographic evidence of SD could only be detected in lateral radiographs.

The OA scores are summarized in Table 2. In 90.8% of affected cases, appendicular OA was bilateral. The elbow joint was most commonly affected and had the largest numbers of osteophytes and increased subchondral radio-opacity. The stifle joint was the next most commonly affected joint, having most evidence of soft tissue swelling, enthesophytosis, and mineralization, with bilateral morbidity present in 85.7% of cases. In the medial meniscus regions of stifle joints affected by OA, 95.9% had evidence of mineralization. Only one owner recognized lameness in one cat with OA in multiple appendicular joints.

**Table 2:** Number of affected joints with each osteoarthritis (OA) score for all appendicular joints.

		**Carpus**	**Elbow**	**Shoulder**	**Hip**	**Stifle**	**Tarsus**
OP	Grade 3	0	5	0	2	0	5
	Grade 2	0	5	0	3	6	6
	Grade 1	3	34	9	16	20	26
	Grade 0	199	158	193	181	176	165
	Number of population	2	25	5	11	14	19
SS	Grade 3	0	0	0	0	0	0
	Grade 2	0	0	0	0	0	0
	Grade 1	0	7	0	0	29	0
	Grade 0	202	195	202	202	173	202
	Number of population	0	4	0	0	15	0
M	Grade 3	0	18	5	3	13	2
	Grade 2	0	7	0	1	15	0
	Grade 1	2	29	4	4	66	6
	Grade 0	200	148	193	194	108	194
	Number of population	2	35	6	4	52	4
OS	Grade 3	0	0	0	0	0	0
	Grade 2	0	0	0	0	0	0
	Grade 1	68	131	57	31	67	86
	Grade 0	134	71	145	171	135	116
	Number of population	34	67	30	18	34	43
E	Grade 3	0	2	0	0	23	11
	Grade 2	0	8	0	0	39	26
	Grade 1	2	35	0	0	40	56
	Grade 0	200	157	202	202	100	109
	Number of population	1	24	0	0	52	48
Total number of affected joints	74	141	69	68	124	114	
Total number of affected cats	37	70	34	36	62	56	

*OP, osteophytes; SS, soft tissue swelling; M, mineralization; OS, osteosclerosis; E, enthesophytes*.

**Figure 2:**
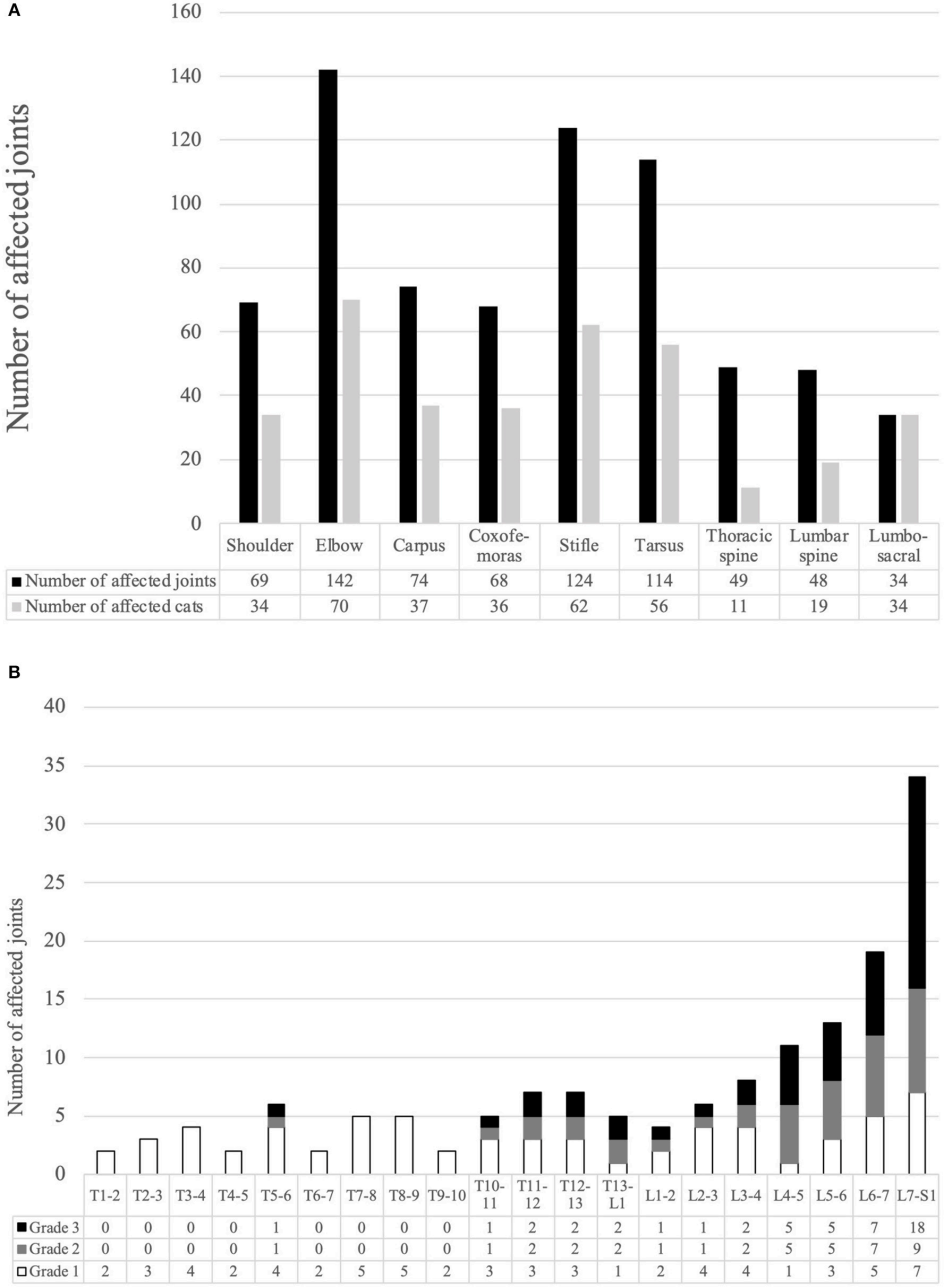
**(A)** Number of affected joints and the number of cats with each degenerative joint disease. **(B)** Number of affected joints with each grade of spondylosis deformans for all intervertebral joint.

The details for SD are shown in Figure 2. Overall, SD of the lumbosacral joint was most common, affecting 34 cats. Grade 3 SD was most common, and seven of the 82 cases with SD in which palpation was possible exhibited obvious back pain on palpation. Only one owner noticed gait abnormality in a cat with SD.

Seven cats (6.93%) had HD, of which five had bilateral dysplasia, with secondary OA noted in these joints. The average NA was 97.46° ± 12.52° in joints without HD and 84.96° ± 6.78° in joints with HD, and the difference was significant. Another 13 cats had transitional vertebrae in a lumbar segment, and three had block vertebrae (two in a thoracic segment and one in a lumbar segment). However, there were no correlations between symptoms and vertebral congenital deformities. A right elbow dislocation and a healed right femoral diaphyseal fracture were seen in 1 cat each, with secondary OA diagnosed in each case.

## Discussion

In this study, we evaluated the use of orthogonal radiographs to diagnose DJD in domestic cats and assessed the prevalence of the condition and the number of radiographs required for diagnosis. Appendicular OA and vertebral SD were common in the domestic short-haired cats from Japan in this study, with a prevalence of DJD comparable to that in previous reports. Radiographic evidence of SD in the lumbosacral joint seemed to have the greatest clinical relevance. Although it may be difficult to diagnose cats with DJD in clinical practice, orthogonal radiography still appears to be the most reliable method when compared to other methods.

The domestic short-haired cats in this study were cats for which the owners required an orthopedic examination. The literature on feline DJD mostly involved populations in which domestic short-haired cats formed the largest component (3–5). Some purebred cats have a proclivity for developing DJD (4). Therefore, the results of this study were considered to reflect feline DJD epidemiology without a breeding bias, even though the data were based on evaluation of a limited feline population in Japan.

In contrast to other reports that failed to show association of OA or SD with sex (2, 10), sex was correlated with appendicular joint OA and intervertebral joint SD, and neutered females had the largest population of DJD-affected cats in the present study (Table 1). When considering the age of the affected group, both those with appendicular OA and SD were significantly older than the healthy group (Table 1). A correlation between sex and DJD was expected, according to the study of Godfrey (5); they had reported that females were significantly more likely to have osteoarthritis. In a human study, women over 65 years of age were the most likely patients of appendicular DJD, especially at the stifle and hip joint (11). The dramatic rise in the prevalence rate of OA after menopause and the presence of estrogen receptors in joint tissues suggest that estrogen may help protect against the development of OA (12). The result of our study showed that DJD was present at age 5.71 ± 6.09 years in females, 12.71 ± 4.99 years in neutered females, 4.55 ± 6.09 years in males, and 8.58 ± 4.81 years in castrated males. Thus, morbidity increased after neutering; the highest level among neutered females may have been related to age. Whether there is sex bias in feline OA needs to be investigated further in future.

The morbidity associated with appendicular OA was different in this study than in previous literature (2–5). For example, OA morbidity increased with increasing age (5), and radiographic evidence of SD showed a different tendency in our study to that seen in previous studies (3, 4). The median ages of the investigated groups in those studies were 15.2 and 6.5 years with corresponding morbidity rates of 80 and 15%, respectively. In this study, the median age of the total cohort was 9.78 ± 5.77 years, and 40.59% of the cohort had morbidity. Both the appendicular OA and vertebral SD populations were significantly older than their respective healthy groups. Therefore, we concluded that acquiring DJD correlated with increasing age in Japanese domestic short-haired cats.

In 90.8% of appendicular OA-affected joints, bilateral symmetry was detected, indicating that OA may be bilateral in felines as a feature. In a previous study, bilateral symmetry was common, and it has been considered to be due to congenital malformations, systemic disease, and overuse (5). The relationship between increased morbidity and age is similar to that of chronic renal failure, among systemic diseases, as reported recently (13). In other reports, inflammatory and immune-mediated etiology has been suggested as the cause of primary DJD in cats (14), and immune dysfunction has been seen in association with DJD in a genomic/proteomic study (15). Some studies have reported the pathology of feline arthritis (16, 17), and it is gradually being elucidated. To further our understanding of the pathophysiology of feline DJD, it is necessary to prove these relationships precisely (18). However, the mechanisms underlying feline DJD have not yet been fully clarified, and further multifaceted investigations will be needed.

The elbow joint was most commonly affected by OA, followed by the stifle joint. A previous study showed a similar trend (5, 19). Most cats with SD had a grade three lesion of the lumbosacral joint. The thoracic vertebrae have previously been reported as the most commonly affected region (4), although another study has emphasized the clinical importance of SD of the lumbosacral joint (3). Although expected, back pain was not noted upon palpation in all but seven cats, which is surprising given that it has been shown to correlate with lumbosacral disease in dogs (20). Recently, the relevance between pain and neurological symptoms with radiographic signs of lumbosacral disease have been reported in cats (3). Performing appropriate diagnostic tests should be considered in a future study of such cats, including the use of magnetic resonance imaging.

Mineralization was detected in the medial menisci of most stifle joints with OA and was also bilateral in most instances. Although the relationship between radiographic evidence of stifle mineralization and histopathological disease has shown a clear association (21, 22), the radiographical evidence and orthopedic examination in this study showed no evidence of anterior cruciate ligament rupture. Therefore, we considered that this feature might be a characteristic of OA among domestic short-haired cats in Japan.

In this study, seven cats (6.93%) had HD, diagnosed radiographically by acetabular depth. The average NA was significantly lower in joints with HD (84.96° ± 6.78°) than in those without HD (97.46° ± 11.52°), and the tendency was comparable to that seen in a prospective study (23). Breed-dependent morbidity has also been revealed by investigating purebred cats (9, 24, 25), but these studies used different HD diagnostic criteria, including assessment of hip joint laxity. Although some congenital vertebral disease was evident in 16 cats in this study, none had associated symptoms. An incidental radiographic finding of congenital vertebral disease may not, therefore, correlate with observed symptoms.

There have been no other reports of feline DJD to date in Asia, but the clinical significance of DJD in vertebral and appendicular joints in this study is consistent with that seen in research conducted elsewhere. However, in previous studies, the radiographic evidence was not only acquired by orthogonal radiographs but also by single-plane radiographs (3–5), except for reports of studies that investigated the use of a questionnaire to assess DJD-associated pain in cats (7, 8, 26–28). Orthogonal radiographic evidence for evaluating DJD was used to support these results (7, 8, 26–28). In general, orthogonal radiography has been recommended for use when diagnosing orthopedic lesions, and only appendicular and vertebral joints were assessed by orthogonal radiography in the present study. Although evaluation of the precise internal joint structure is difficult based on a single-plane radiograph (17), the utility of the single radiograph for evaluation of joint incongruity has been reported (29) in a human study. Appendicular OA, especially the hip and stifle joints, probably requires two radiographic views for diagnosis.

A recent report has shown that gait analysis is useful clinically (30), and it was reported that questioning owners could uncover various potentially useful signs for detecting and monitoring OA (31). In our study, only one owner recognized symptoms related to SD and one recognized symptoms related to appendicular OA, similar to previous reports (3, 4). A simple questionnaire-based tool, by which the owner assesses changes in their cat's behavior/lifestyle, has been useful for identifying chronic musculoskeletal pain in their pets (32). Although these methods may detect the possibility of morbidity of feline OA, orthopedic diagnosis and localization still depends on radiography in cats (33).

Accordingly, an increasing number of radiographs are used to diagnose feline orthopedic problems, which increases exposure to radiation. Three-dimensional diagnostic tools, for example computed tomography and magnetic resonance imaging, may have an increased possibility to detect the region on appendicular OA or SD; despite this, the patient receiving a screening examination that uses computed tomography must be exposed to large amount of radiation for it. According to our result, radiographic evidence of SD was only detectable in lateral radiographs, and our findings might justify reducing the number of radiographs taken to diagnose SD, but this needs further investigation.

A previous study revealed the role of articular process assessment on vertebrae using radiographic evaluation in humans (34). Therefore, we investigated the potential of evaluating articular processes on orthogonal radiographs in cats, but this was found to be difficult, especially in obese cats, because the density around articular process is affected by variation of thickness in cats (body condition score). In human studies, three-dimensional imaging devices have been used for precise evaluation of articular processes and revealed a correlation between disc and facet joint degeneration (35, 36) and patients' symptoms. In contrast, it is difficult to identify symptoms in cats, as these are dependent on the owner's attentiveness, and the difficulty was confirmed in our experience. Ultrasound evaluations have drawbacks that include time requirements. Therefore, for precise vertebral evaluations, we consider that three-dimensional evaluations are more appropriate.

## Data Availability Statement

The raw data supporting the conclusions of this article will be made available by the authors, without undue reservation, to any qualified researcher.

## Ethics Statement

Ethical review and approval was not required for the animal study because this research was a summary by clinical findings which are a part of diagnosis and treatment in an animal hospital, and we got approvals of conducting this study and making a report from all owners of cats. Written informed consent was obtained from the owners for the participation of their animals in this study.

## Author Contributions

TKim was responsible for the assessment of radiographs, the data analysis, conception of the study, and manuscript writing and revisions. TKit drafted the manuscript and gave approval for the submitted manuscript. SK, SS, and JO were responsible for acquiring the radiographs. All authors had access to the study data and made the final decision on the submission of these data.

### Conflict of Interest

The authors declare that the research was conducted in the absence of any commercial or financial relationships that could be construed as a potential conflict of interest.

## Abbreviations

BCS: body condition score
DJD: degenerative joint disease
HD: hip dysplasia
NA: Norberg angle
OA: osteoarthritis
SD: spondylosis deformans.
